# Deciphering intra-species bacterial diversity of meat and seafood spoilage microbiota using *gyrB* amplicon sequencing: A comparative analysis with 16S rDNA V3-V4 amplicon sequencing

**DOI:** 10.1371/journal.pone.0204629

**Published:** 2018-09-25

**Authors:** Simon Poirier, Olivier Rué, Raphaëlle Peguilhan, Gwendoline Coeuret, Monique Zagorec, Marie-Christine Champomier-Vergès, Valentin Loux, Stéphane Chaillou

**Affiliations:** 1 MICALIS, INRA, AgroParisTech, Université Paris-Saclay, Jouy-en-Josas, France; 2 MaIAGE, INRA, Université Paris-Saclay, Jouy-en-Josas, France; 3 Secalim, INRA, Oniris, Nantes, France; Agricultural University of Athens, GREECE

## Abstract

Meat and seafood spoilage ecosystems harbor extensive bacterial genomic diversity that is mainly found within a small number of species but within a large number of strains with different spoilage metabolic potential. To decipher the intraspecies diversity of such microbiota, traditional metagenetic analysis using the 16S rRNA gene is inadequate. We therefore assessed the potential benefit of an alternative genetic marker, *gyrB*, which encodes the subunit B of DNA gyrase, a type II DNA topoisomerase. A comparison between 16S rDNA-based (V3-V4) amplicon sequencing and *gyrB*-based amplicon sequencing was carried out in five types of meat and seafood products, with five mock communities serving as quality controls. Our results revealed that bacterial richness in these mock communities and food samples was estimated with higher accuracy using *gyrB* than using16S rDNA. However, for *Firmicutes* species, 35% of putative *gyrB* reads were actually identified as sequences of a *gyrB* paralog, *parE*, which encodes subunit B of topoisomerase IV; we therefore constructed a reference database of published sequences of both *gyrB* and *pare* for use in all subsequent analyses. Despite this co-amplification, the deviation between relative sequencing quantification and absolute qPCR quantification was comparable to that observed for 16S rDNA for all the tested species. This confirms that *gyrB* can be used successfully alongside 16S rDNA to determine the species composition (richness and evenness) of food microbiota. The major benefit of *gyrB* sequencing is its potential for improving taxonomic assignment and for further investigating OTU richness at the subspecies level, thus allowing more accurate discrimination of samples. Indeed, 80% of the reads of the 16S rDNA dataset were represented by thirteen 16S rDNA-based OTUs that could not be assigned at the species-level. Instead, these same clades corresponded to 44 *gyrB*-based OTUs, which differentiated various lineages down to the subspecies level. The increased ability of *gyrB*-based analyses to track and trace phylogenetically different groups of strains will generate improved resolution and more reliable results for studies of the strains implicated in food processes.

## Introduction

Effective management of our food chain is strongly dependent on a thorough understanding of microbial ecology, which has applications in elucidating contamination routes, controlling microbial food spoilage, predicting shelf life accurately, and improving food safety. In particular, improved precision in identifying the species and/or strains implicated in the microbial spoilage of meat and seafood could contribute to a better understanding of microbial ecology far beyond the spoilage mechanism. Bacteria are the predominant spoilage micro-organisms on meat and seafood, but the spoilage microbiota is usually not very complex: between 15 to 80 species, on average, with the population abundances of dominant and subdominant strains typically differing by four orders of magnitude [1–5]. This microbiota also has limited phylogenetic diversity, as most species belong to the phyla *Firmicutes* (orders *Lactobacillales*, *Bacillales*) or *Proteobacteria* (orders *Enterobacterales*, *Pseudomonadales*, and *Vibrionales*) among which a large majority is known and cultivable under laboratory conditions. Yeasts and molds are also present in lower proportions, but are rarely monitored.

Despite their importance, we still understand very little about the actual roles played by the spectrum of bacterial species involved in spoilage. Spoilage development is an intricate biological phenomenon, which can be species- or even strain-specific [6, 7]. Certain microbial taxa may be influenced differently by a given set of storage conditions, and some microbial species may develop unpredictably during meat storage, which together influence the time and type of spoilage [8]. Consequently, more information is required in order to assess, within each spoilage group, which species or intra-species lineages are actually involved in the spoilage of food products [9, 10]. Strain’s variability arises from differences in food origins, routes of contamination, and production processes of the food itself, but also in the variety of packaging and storage methods used. These factors, and the interactions among them, have the potential to generate a wide diversity of bacterial strains through selective pressures. Furthermore, many food-borne bacteria that are involved in spoilage also display broad genomic diversity at the intraspecies level [11–15]. Within a given species, certain strains may carry key gene clusters involved in the fitness or adaptation to specific conditions or in the production of compounds important for food fermentation or spoilage while other strains do not. To analyze such functional genomic diversity, shotgun metagenomic analysis is of course the most powerful approach. However, this strategy requires extensive sequencing efforts to capture the genetic diversity of sub-dominant populations, whose abundances can differ by several orders of magnitude. Furthermore, it is not cost effective in the context of the large sampling campaigns often required to study fluctuations in food microbial diversity over the course of a production process. To address these limitations, it has become common to use 16S-rDNA based amplicon sequencing (also called metagenetic analysis) and then, based on the species identified, infer some of the functions carried out by the microbiota with tools like PICRUST [16] or Tax4Fun [17].

Despite the extraordinary insights that have been gained through 16S rDNA profiling analyses, taxonomic methods based on this approach have several shortcomings, particularly at the shallowest taxonomic levels. The extremely slow rate of evolution of this gene hinders the resolution of closely related bacteria into individual 16SrDNA phylotypes. In particular, the practice of clustering OTUs at 97% (or even 99%) 16S rDNA sequence identity will group together functionally diverse lineages, thus concealing significant amounts of species- and strain-level variation. Moreover, variation in the number of rRNA operons among different bacterial species creates problems for the quantification of cell numbers or taxon abundances based on 16S rDNA phylotypes [18, 19]. Biases in quantification also arise from chimera formation due to the highly conserved sequence of the gene. The resulting artificial sequencing errors then inflate estimates of OTU diversity. This marker, specifically the rDNA V1-V3 region, has been used in many studies of food microbiota. Its popularity was based on the presence of three variable regions at the 5’ end of the 16S rRNA gene, which offered sufficient variability with which to discriminate among species of lactic acid bacteria (order *Lactobacillales*), one of the bacterial groups that is predominant in food. However, analysis of this region required long sequencing reads (>550 bp), which was only possible with pyrosequencing technology. Recently, however, a more-accurate technology (Miseq pair-end sequencing) has come into favor because it generates increased sequencing depth. As a result, studies have shifted their focus to the 16S rDNA V3-V4 region, because this region is shorter (~450 bp) and thus promotes improved merging of forward and reverse reads. Unfortunately, the 16S rDNA V3-V4 region does not offer the same discriminatory power as the V1-V3 region for identifying species-level diversity. The traditional 97% identity threshold used for OTU clustering does not adequately resolve species diversity when only a few variable regions of the 16S gene are amplified; indeed, depending on the regions used, results may vary widely. For these reasons, 16S rDNA amplicon data are often analyzed at the genus level only, but these results lack the power to yield informative answers to many questions, including those mentioned in the previous paragraph. Because of this, we sought an alternative marker that could improve diversity analysis at the species- or even intraspecies-level while keeping the ease-of-use and cost-effectiveness of amplicon sequencing.

To address this type of problem, some studies (not dedicated to food microbiota) have ventured beyond the analysis of 16S sequences by targeting coding regions with conserved primers or by extracting coding-gene orthologs from shotgun metagenomics surveys [20–22]. Indeed, advances in molecular microbial ecology have opened avenues for the design of taxonomically meaningful, highly specific PCR primers. Protein-coding genes, which evolve much faster than the 16S rRNA gene, are useful for differentiating among more-recently diverged lineages, but their application is complicated by difficulties in designing low-redundancy primers that amplify homologous regions from distantly related taxa. Thus, there is no broadly applicable community-profiling method based on protein-coding genes that is analogous to those based on rRNA genes. One difficulty in devising such a method stems from the high variability of protein-coding genes, particularly at synonymous codon positions, which thwarts the design of universally conserved primers. Additionally, bacterial lineages vary in their genomic contents, which suggests that different genes might be needed to resolve the diversity within certain taxonomic groups. The genes that have been proposed for this task include those encoding 23S rRNA, DNA gyrase subunit B (*gyrB*) [21, 23, 24], RNA polymerase subunit B (*rpoB*) [25], TU elongation factor (*tuf*) [26], DNA recombinase protein (*recA*), protein synthesis elongation factor-G (*fusA*), and dinitrogenase protein subunit D (*nifD*) [27]. An ideal candidate should contain well-conserved regions to facilitate the design of primers and molecular probes to be used in the identification of food microbiota.

Among these, *gyrB* has a higher rate of base substitution than 16S rDNA does, and shows promise for community-profiling applications [28–30]. This gene is essential and ubiquitous in bacteria and is sufficiently large in size for use in analysis of microbial communities [31]. It is a single-copy housekeeping gene that encodes the subunit B of DNA gyrase, a type II DNA topoisomerase, and therefore plays an essential role in DNA replication. Furthermore, the *gyrB* gene is also present in *Eukarya* and sometimes in *Archaea* but it shows enough sequence dissimilarity between the three domains of life to be used selectively for Bacteria [32].

The main objective of the current work was to validate the usefulness of *gyrB* as an alternative phylogenetic marker to accurately and precisely discriminate closely related species within various food microbiota. We therefore carried out a comparison of amplicon sequencing based on 16S rDNA V3-V4 and that based on *gyrB* using five types of meat and seafood products (pork sausage, poultry sausage, cod filet, salmon filet, and ground beef). These products were specifically chosen because their microbiota have been extensively studied [1]and comprise a broad spectrum of bacterial species from the phyla *Firmicutes* and *Proteobacteria*. In order to assess the added value brought by *gyrB* sequencing with respect to 16S rDNA sequencing, five mock communities (MC) were constructed as quality controls, using 15 different species with a high degree of intraspecies diversity.

## Materials and methods

### Preparation of mock bacterial communities

Bacterial strains were grown overnight at 20°C in 5 ml of either MRS broth (*Lactobacillus*, *Leuconostoc*), M17 + 0.5% w/v of glucose broth (*Lactococcus*), or BHI broth (*Carnobacterium*, *Brochothrix*, *Serratia*, *Pseudomonas*). The cell concentration of each culture was checked by measuring the optical density at 600 nm. Cells were pelleted by a 10-min centrifugation at 3,000 x g at 4°C and washed with sterile deionized water. After a second centrifugation at 3,000 x g, cells were re-suspended in sterile water so as to obtain a concentration of 10^9^ cells.ml^-1^. Mock communities were then created by mixing the different strains at the desired concentration ratio. One milliliter of the mock community mixture was collected for DNA extraction. Mock community 5 (MC5), which was of unknown bacterial composition, was obtained by mixing the bacterial pellets obtained after the extraction (see next paragraph) of eight different food products (ground beef, pork fillet, lamb fillet, turkey fillet, salmon fillet, cod fillet, whiting fillet, and rainbow trout fillet) analyzed on the use-by date. Details of the composition of these bacterial MCs are presented in Table 1.

**Table 1:** Bacterial composition of mock communities (in percentage of each taxon^a^).

Phyla& their associated Bacterial species	Strains	Reference of strains	Mock communities			
			Inter-species			Intra-species
			MC1	MC2	MC3	MC4
***Firmicutes***	-		99%	50%	1%	65%
***Proteobacteria***			1%	50%	99%	35%
**Composition within the *Firmicutes* (summed up to 100%)**						
*Lactobacillus algidus*	CMTALT10	[33]	5%			-
*Lactobacillus sakei*	23K	[34]	35%			20%
	DSM 20017	[35]	-			20%
	DSM15831	[35]	-			20%
*Lactococcus piscium*	CMTALT02	[33]	10%			
*Brochothrix thermosphacta*	160x8	[33]	5%			10%
	cH814	[36]	-			5%
	ATCC11509	[37]	-			5%
*Carnobacterium divergens*	MFPA43A14-05	[33]	10%			-
*Carnobacterium maltaromaticum*	DSM20342	[35]	10%			-
*Leuconostoc gelidum subsp*. *gasicomitatum*	MFPA44A14-01	[33]	5%			15%
*Leuconostoc gelidum subsp*. *gelidum*	DSM5578	[38]	-			5%
*Weissella viridescens*	MFPC16A28-05	[33]	20%			-
**Composition within the *Proteobacteria* (summed up to 100%)**						
*Pseudomonas fragi*	ATCC4973	[39]	20%			-
*Pseudomonas lundensis*	MFPA15A12-05	[33]	15%			20%
	MFPB42A12-09	[40]	-			10%
	PCAi D2.2	[41]	-			10%
*Acinetobacter guillouiae*	MFPA43A14-04	[40]	5%			-
*Photobacterium phosphoreum*	CIP105612	[42]	25%			-
*Serratia proteamaculans*	MFPA44A14-05	[43]	10%			20%
	1C2F	[36]	-			20%
	CIP 103236	[44]	-			20%
*Hafnia alvei*	CIP57.31	[45]	5%			-
*Morganella psychrotolerans*	MFPA43A14-03	[40]	20%			-

^a^Taxon percentages are first given comparatively for *Firmicutes* and *Proteobacteria*. Within each phylum, the percentages are then given for each taxon.

### DNA extraction and barcoding PCRs for MiSeq sequencing

#### DNA extraction of the bacterial microbiota

Ten grams of each food-product batch were homogenized in 40 ml of sterile ultrapure water supplemented with 1% Tween 80 (Acros Organics, Waltham, USA) for 30 s in a stomacher. Then, 32 ml of the shreds were collected and centrifuged at 500 × g for 3 min at 4°C to spin down the food matrix fibers and debris. The still-turbid supernatant (~25 ml) was collected and centrifuged at 3,000 × g for 5 min at 4°C to spin down the bacterial cells. The bacterial pellet thus obtained was washed in 1 ml of sterile ultrapure water and collected after centrifugation at 3,000 × g for 5 min at 4°C to serve directly for DNA extraction or to compose MC5 for further DNA extraction.

To minimize potential biases associated with the DNA extraction method, bacterial DNA from all samples was extracted according to the manufacturer’s instructions with two different kits: the PowerFood Microbial DNA Isolation kit (MoBio Laboratories Inc., Carlsbad, USA) and the High Pure PCR Template Preparation kit (Roche Diagnostics Ltd, Burgess Hill, West Sussex, UK). For each sample, both DNA extracts were pooled.

#### Purification and quantification of initial 16S rDNA (V3-V4) and *gyrB* PCR

Amplicon libraries were constructed following two rounds of PCR amplification. The first amplification of the ~450-bp V3-V4 hypervariable regions of the bacterial 16S rRNA gene was performed with the primers V3F (5’-ACGGRAGGCWGCAGT-3’) and V4R (5’-TACCAGGGTATCTAATCCT-3’)[46]. In parallel, the degenerate primers F64 (5’-MGNCCNGSNATGTAYATHGG-3’) and R353 (5’-CNCCRTGNARDCCDCCNGA-3’) were used to amplify a ~280-bp region of *gyrB*. The primers’ binding sites correspond to *Escherichia coli* E22 (IMG taxon ID, 638341087) nucleotide positions 64 to 353 as described in[21]. Forward and reverse primers carried the Illumina 5’-CTTTCCCTACACGACGCTCTTCCGATCT-3’ and the 5’-GGAGTTCAGACGTGTGCTCTTCCGATCT-3’ tails, respectively. The first round of PCRs was performed with two different high-fidelity polymerases: Moltaq 16S (Molzym Life Science, Bremen, Germany) for the 16S V3-V4 region and the AccuPrime Taq DNA polymerase system (Invitrogen, Carlsbad, USA) for *gyrB*, using in both cases the manufacturer’s protocol and 2 μL of microbial DNA (approximately 10 ng). The cycling conditions for the V3F/V4R 16S reaction mixtures were: 94°C for 1 min, followed by 30 cycles of amplification at 94°C (60 s), 65°C (60 s), and 72°C (60 s), with a final extension step of 10 min at 72°C. Amplification of *gyrB* was performed as follows: 94°C (2 min) followed by 35 cycles of amplification at 94°C (30 s), 55°C (60 s), and 68°C (90 s), with a final extension step of 10 min at 68°C. For the V3-V4 region, the final primer concentration used was 200 nM, whereas for *gyrB*, the final primer concentration was increased to 1000 nM to compensate for the high degeneracy of the primers. All PCRs were performed in triplicate. Replicates were pooled and the amplified DNA was purified with a QIAquick kit (Qiagen, Hilden, Germany). Amplicon size, quality, and quantity were checked on a DNA1000 chip (Agilent Technologies, Paris, France).

#### Purification and quantification of the second Illumina Miseq PCR

In the second round of PCR, sample multiplexing was performed by adding tailor-made 6-bp unique index tags to the ends of the forward and reverse adapters (5’-AATGATACGGCGACCACCGAGATCTACACT-3’ and 5’-CAAGCAGAAGACGGCATACGAGAT-NNNNNN-GTGACT-3’, respectively). This second PCR step was performed on 50–200 ng of purified amplicons from the first PCR using 2.5 U of a DNA-free Taq DNA Polymerase and 1xTaq DNA polymerase buffer. The buffer was composed of 10 nmol of dNTP mixture (Sigma-Aldrich, Saint-Louis, USA), 25 nmol of each primer (Eurofins, Luxembourg, Luxembourg), and nuclease-free water (Qiagen, Hilden, Germany) up to a final volume of 50 μl. The reaction was carried out on a T100 thermal cycler with an initial denaturation step (94°C for 10 min), 12 cycles of amplification (94°C for 1 min, 65°C for 1 min, and 72°C for 1 min), and a final elongation step at 72°C for 10 min. Amplicons were purified using Clean PCR magnetic beads (CleanNA, Alphen aan den Rijn, The Netherlands) in a 96-well format. The concentration of the purified amplicons was measured using a Nanodrop spectrophotometer (Thermo Scientific, Waltham, USA) and the quality of a subset of amplicons (12 samples per sequencing run) was controlled on a Fragment Analyzer (AATI, Santa Clara, USA) with the ADNdb 910 reagent kit (35–1,500 bp). Controls were included to ensure that the high number of PCR cycles (35 cycles for PCR1 + 12 cycles for PCR2) did not create significant amounts of PCR chimeras or other artifacts. Negative controls were also included; these used nuclease-free water (Qiagen, Hilden, Germany) in place of the extracted DNA during the library preparation. All libraries were pooled using equal amounts in order to generate the equivalent number of raw reads for each library. The DNA concentration of the pool (no dilution, diluted 10x or25x in EB + 0.5% Tween buffer) was quantified on a Qubit Fluorometer (Thermofisher Scientific, USA). The final pools used for sequencing had a concentration between 5 and 20 nM.

#### Illumina Miseq sequencing

The pool was denatured (NaOH 0.1N) and diluted to 7 pM. PhiX Control v3 (Illumina, San Diego, USA) was added to the pool at 4.5% of the final concentration. From this mixture, 600 μl were loaded onto the Illumina MiSeq cartridge according to the manufacturer’s instructions using the MiSeq Reagent Kit v3 (2x300 bp paired-end reads, 15 Gb output). FastQ files were generated at the end of the run (MiSeq Reporter software, Illumina, USA) for quality control. The quality of the run was checked internally using PhiX Control and then each paired-end sequence was assigned to its sample of origin using the multiplexing index tag. Raw read sequences were deposited at the Sequence Read Archive under the accession numbers SAMN09070427to SAMN09070506.

### Quantification of target species using real-time PCR

Real-time PCR quantification of 13out of the 16 target species added in the mock communities was carried out on all food samples and mock communities using a set of specific probes previously published by our team [47] and described in S1 Table. The qPCR amplifications were performed on a RealPlex thermal cycler (Eppendorf, Hamburg, Germany). All analyses were performed in duplicate using a 10-fold dilution of the DNA extracts to avoid inhibitor effects. The reaction mixture consisted of 10 μM of each primer (forward and reverse), 10 μl of SYBR MESA GREEN master mix reagent kit (Eurogentec, Liège, Belgium), and 4.2 μl of sterile water. A total of 15 μL of this mixture was added to 5 μL of DNA extract. Each PCR run included a positive control isolated from type strains of each targeted species, and a negative control. The qPCR amplification started with one cycle at 95°C for 2 min, followed by 40 cycles of denaturation at 95°C for 15 s and annealing at 60°C for 1 min. The step for melting-curve checking was performed at 95°C for 15 s, 1 min at 60°C, and 20 min ramp from 60°C to 95°C. Average threshold cycle (C_T_) was calculated for each pair of samples. The population level of the target bacteria was estimated in CFU.g^-1^ of food sample. Two equations were used (Eqs 1 and 2 below), depending on whether the gene probe was based on the 16S rRNA gene or on another housekeeping gene, respectively.

$${CFU.g}^{-1}=\frac{e^{C_{t}-39.43}}{-1.52}$$ (1)

$${CFU.g}^{-1}=\frac{e^{C_{t}-40.98}}{-1.44}$$ (2)

These equations were obtained from several independent biological replicates of calibration curves, which were carried out as described previously [47]. To convert the relative number of reads obtained for a given species in each sample (*nr*) into an absolute number of reads normalized by the total bacterial cell concentration in that sample (*na*), Eq 3 was applied.

$$na=\frac{nr\times Q}{Nt}$$ (3)

where *Q* is the total concentration of bacterial cells in the sample in CFU.g^-1^ as obtained by qPCR using the all_bacteria primers (see S1 Table), and *Nt* is the normalized sum of reads in the sample.

Based on these results, a linear model was constructed using the *lm* function in R. For each species, we calculated the deviation between the experimental number of reads and the expected value obtained with the linear model in order to compare amplicon quantification and quality. Positive deviations were attributed to an overestimation of the relative abundance of a given species due to sequencing bias, while negative deviations were associated with an underestimation of the proportion of the species within the ecosystem.

### Quality filtering, definition of OTUs, and taxonomic assignment

The quality of the sequencing was first evaluated using FastQC [48]. Individual reports were merged into a single one with MultiQC [49]. The 16S rDNA and *gyrB* paired-end sequences were merged into contigs with PEAR v0.9.10 [50]. Adapters were trimmed with cutadapt v1.12 [51]. Low-quality bases at the extremities of sequences were removed using Sickle v1.330 [52]. Data were subsequently imported into the FROGS (Find Rapidly OTUs with Galaxy Solution) pipeline [53]. Sequences were dereplicated before being clustered using SWARM [54] with a local clustering threshold with a distance of 3. Chimeras were removed with vsearch [55]. The resulting sequences were filtered for spurious OTUs likely arising from sequencing artifacts (OTUs with low abundance and low frequency) by keeping only those appearing more than 10 times in the whole dataset [56]. Taxonomic assignment of OTUs that corresponded to 16SrRNA sequences was performed using Silva 128 SSU [57] as reference database, while a homemade databank was created for the *gyrB* and *parE* sequences (see below), using in both cases the Blastn+ algorithm[58]. Our Bioinformatic report for these initial steps can be found here: http://genome.jouy.inra.fr/analyses/REDLOSSES-gyrB/report.html.

### Construction of *gyrB* and *parE* database

A total of 44,494 genomes were downloaded from Ensembl Bacteria (release 38, January 2018) [59]. Then, embl files were parsed with a BioPython library to obtain only sequences of *gyrB* and *parE* in FASTA format. To be sure we kept only sequences of interest, we processed all sequences with cutadapt v1.12 [51]. If 5' or 3' extremity primers were not found, the sequences were discarded. The resulting 44,572 *gyrB* and 30,549 *parE* sequences were combined with another 30,525 sequences extracted from IMG (release 4.3) and with 389 additional sequences from strains and species known to be important in food microbiota that were not represented in Ensembl and IMG [60]. The final database contained 106,035 sequences which were submitted to FROGS analysis for taxonomic assignment.

### Analysis of alpha and beta diversity

Bacterial diversity was analyzed using the R package Phyloseq [61]. To facilitate comparative analysis between 16S rDNA data and *gyrB* data, a common Phyloseq object was created which comprised a single otu_table, sample_data, and tax_table (the whole dataset is available at (DOI:10.6084/m9.figshare.7083209). OTU abundance was normalized using the median sequencing depth of all samples (for both 16S rDNA and *gyrB*). Analyses of alpha and beta diversity were then carried out using standard or custom Phyloseq command lines. Our R script (redlosses_phyloseq_custom.R), which includes all commands performed to create our figures, is available for download at (DOI:10.6084/m9.figshare.7083254).

### Construction of phylogenetic trees

Complete sequences of *gyrB* and *parE* genes were extracted from the genome database. They were trimmed with cutadapt v1.12 in order to keep only sequences that were amplified by the degenerate primers. The phylogenetic trees were constructed by nucleotide alignment, using the Kimura 2-parameter algorithm and the neighbor-joining method implemented in MEGA 6 software [62].

## Results

### Study design and sample collection

#### Food samples

Five different food products (ground beef burgers, pork sausages, poultry sausages (turkey), cod fillets, and salmon fillets), all packaged under modified atmosphere, were selected for this study. For each food item, three batches (biological replicates) were purchased in supermarkets one week apart and stored at 8°C until the product’s use-by date. The nomenclature of the food samples and their associated dataset items are described in Table 2. These products were chosen because the composition of their microbiota has already been well described [1, 47]. In addition, the different communities offered a good system with which to study variations in abundance between the two main bacterial phyla (*Firmicutes* and *Proteobacteria*) and the various species therein.

**Table 2:** Food and mock sample names and sequencing dataset nomenclature.

Sample type	Sample name	16S dataset name	*gyrB* dataset name
Cod fillets	CF1 to CF3	*e*.*g*. CF1_16S	*e*.*g*. CF1_GYRB
Salmon fillets	SF1 to SF3	*e*.*g*. SF1_16S	*e*.*g*. SF1_GYRB
Ground beef burgers	GB1 to GB3	*e*.*g*. GB1_16S	*e*.*g*. GB1_GYRB
Poultry sausages	CS1 to CS3	*e*.*g*. CS1_16S	*e*.*g*. CS1_GYRB
Pork sausages	PS1 to PS3	*e*.*g*. PS1_16S	*e*.*g*. PS1_GYRB
Mock communities	MC1 to MC5	*e*.*g*. MC1_16S	*e*.*g*. MC1_GYRB

#### Mock communities

In order to assess the benefits of *gyrB* amplicon sequencing compared to that of 16S rDNA, five mock communities (MC) were constructed as quality controls. Three of these (MC1, MC2, MC3) consisted of 15 strains belonging to 15 genetically diverse bacterial species which were mixed at different cell concentrations; species belonged to the two main phyla (*Firmicutes* and *Proteobacteria*) that are commonly recovered in the food products used as test samples. Briefly, MC1 consisted of 99% *Firmicutes* and 1% *Proteobacteria*, MC2 contained 50% *Firmicutes* and 50% *Proteobacteria*, and MC3 was composed of 1% *Firmicutes* and 99% *Proteobacteria*. The fourth mock community (MC4) was designed to assess the species-level or even intraspecies-level accuracy of taxonomic assignment. It comprised14strains that have been completely sequenced and are affiliated with five of the bacterial species previously selected for MC1-3. The fifth mock community (MC5) of unknown complex composition was designed to maximize overall alpha diversity: it was created by mixing the bacterial pellets extracted from eight different food products. With this MC, we wanted to compare the relative abilities of the two marker genes to accuracy capture a high level of species diversity.

### Bacterial richness

**Fig 1:**
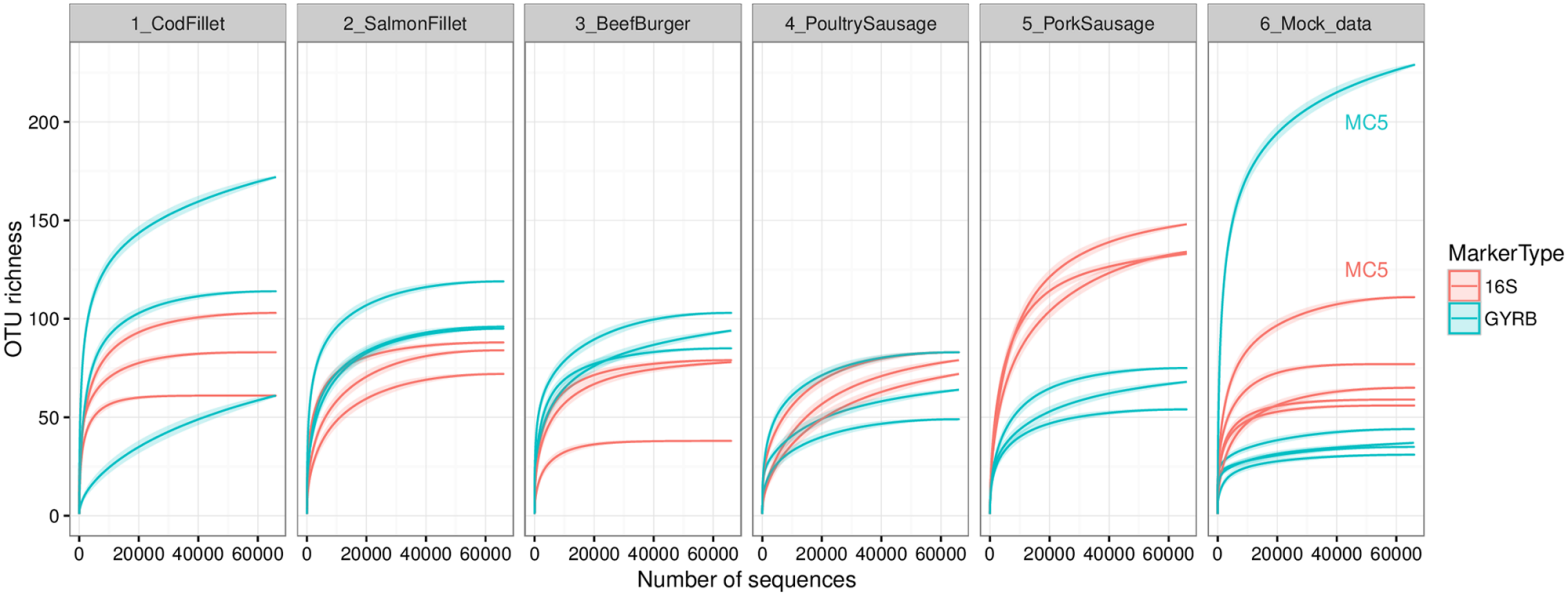
Rarefaction curves obtained from16S rDNA and *gyrB* amplicon sequencing of the three repeats for each food sample and the five mock communities. The x-axis represents the sequencing depth in number of reads while the y-axis represents an estimation of the OTU richness detected. Samples are presented separately, with each panel representing data from one food type or the mock communities. Rarefaction curves for the MC5 community are specifically indicated on the far-right panel.

Food samples and mock communities were sequenced at an average of 66,012 ± 21,134 reads. Rarefaction curves (Fig 1) performed on quality-filtered reads indicated that sequencing depth was sufficient for all samples, including, notably, the complex mock community MC5. Bacterial OTU richness (Table 3) were comparable between the two markers only for four product types: salmon fillet, cod fillet, ground beef burger, and poultry sausage. For pork sausages, the two markers yielded significantly different results. The most striking difference was found in the bacterial OTU richness obtained from pork sausages, which was much higher when analyzed with the 16S rDNAV3-V4 region than with *gyrB*. Interestingly, the results from the three biological replicates (batches) of cod fillet were very different from each other, and this difference was even more pronounced in the *gyrB* analysis than in the 16S rDNA analysis. When we merged OTUs into genus-level assignments and re-estimated richness (Table 3), the two markers gave much more consistent results, but again with the exception of the pork sausage samples.

**Table 3:** Comparison of bacterial richness between 16S rDNA and *gyrB* amplicon sequencing.

Sample description	Number of observed OTUs (Species richness)		Diversity of genus-level taxa (Genus richness)	
	16S	*gyrB*	16S	*gyrB*
Cod fillets (CF)	82 ± 14	117 ± 36	26 ± 06	28± 06
Salmon fillets (SF)	81 ± 06	103 ± 36	23 ± 02	25 ± 01
Ground beef burgers (GB)	65 ± 18	94 ± 06	20 ± 04	19 ± 03
Poultry sausages (CS)	78 ± 04	65 ± 11	15 ± 01	13 ± 01
Pork sausages (PS)	138 ± 06	66 ± 06	40 ± 02	13 ± 02
Mock MC1 (15 species)	59	42	15	15
Mock MC2 (15 species)	77	53	16	15
Mock MC3 (15 species)	56	37	15	14
Mock MC4 (5 species)	65	50	8	5
Mock MC5 (complex community)	110	229	33	40
The whole dataset	331	369	64	51

Similarly, OTU richness in mock communities MC1 and MC4 was higher in the 16S analysis than in that based on *gyrB*; the latter gene yielded values that were closer to the number of different species and genera that we had expected to be detected (*n* = 15). For mock community MC5, its unique composition (combined bacterial community from eight different food products) was expected to generate a high diversity of species and strains. Based on previous data from these types of products [1], we had estimated that MC5 would contain between 150and 200 different species. However, it appeared that the 16S rDNA-based analysis underestimated the richness of this bacterial assemblage, particularly in light of the results obtained for MC1 and MC4. Based on these initial results, the *gyrB* marker seemed to be more sensitive than 16S for the purpose of estimating bacterial richness. We hypothesized that this result might be associated with an increased ability of *gyrB* to detect species-level or intraspecies-level diversity.

### Both *gyrB* and *parE* genes are amplified within *Firmicutes*

**Fig 2:**
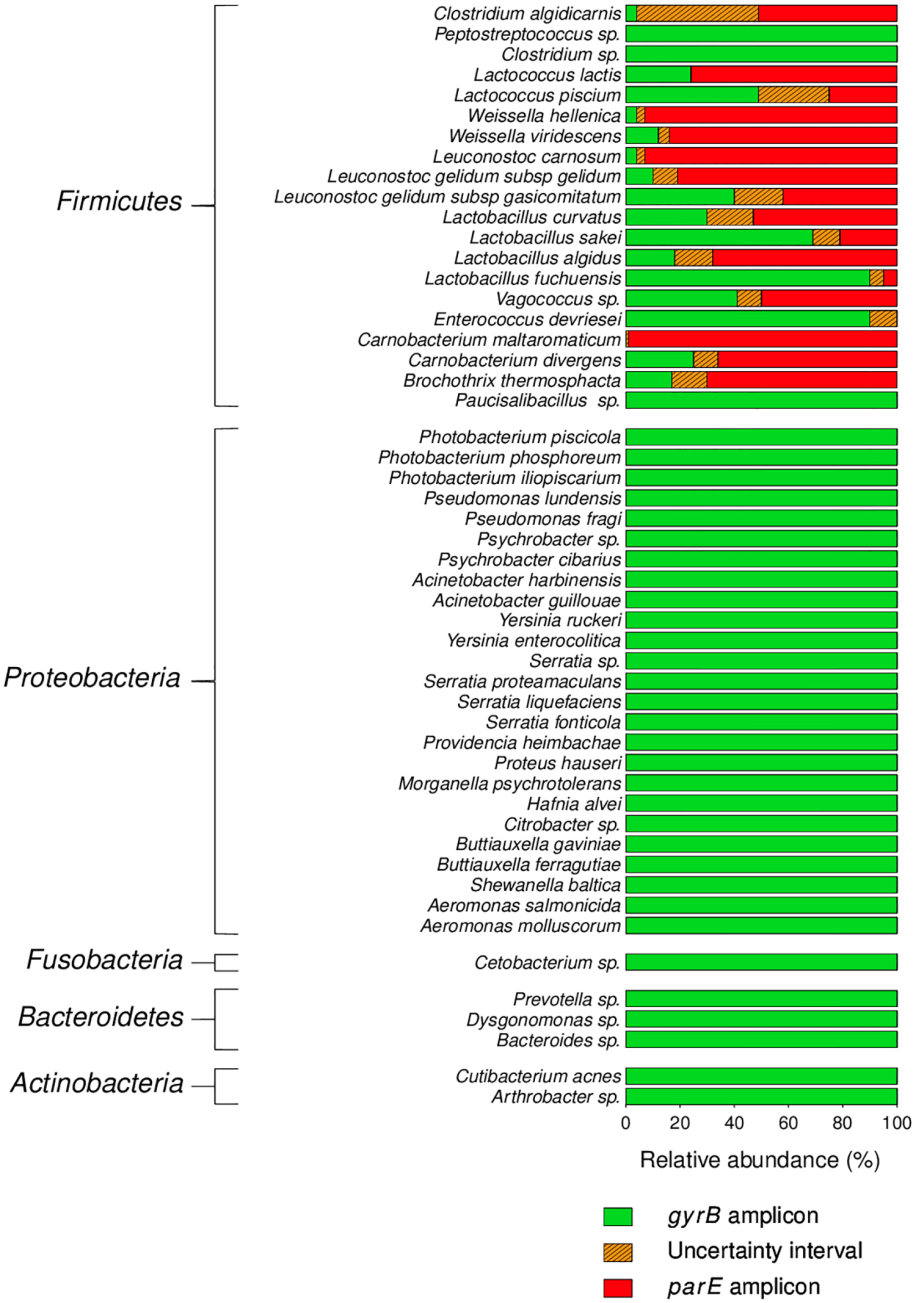
Relative abundance of *gyrB* and *parE* sequences assigned to the fifty most-abundant genera recovered in the food samples and mock communities. The limits of the uncertainty intervals correspond to the upper and lower standard deviation of the average proportion of *gyrB* reads obtained across the various samples in which each taxon was detected.

In order to assign taxonomic identifications to OTUs obtained with *gyrB* sequencing, we constructed a *gyrB* database (see Materials& methods section). However, our first attempt at assignment revealed that 72 OTUs within the whole g*yrB* dataset were not assigned to any species; these OTUs accounted for 19.5% of the total diversity and 35% of the total read in the dataset. Further investigation revealed that these OTUs demonstrated affinity to sequences of *parE*. The *parE* gene encodes subunit B of topoisomerase IV, an enzyme involved in chromosome segregation in bacteria (Interpro accession IPR005740). Because the ParE and GyrB proteins are considered to be paralogs, their respective genes may show significant sequence similarity. To check whether the confusion between *parE/gyrB* occurred for all types of taxa, we analyzed the proportions of *gyrB* and *parE* sequences assigned to the fifty most-abundant genera from our dataset (Fig 2). Within four of the five phyla recovered in all samples—i.e. *Proteobacteria*, *Bacteroidetes*, *Actinobacteria*, and *Fusobacteria*—all amplicon sequences were specifically assigned to *gyrB*. This result indicated that among these phyla, the *parE* gene sequence was sufficiently dissimilar from the *gyrB* sequence as to not be co-amplified. Instead, within *Firmicutes*, both genes were recovered at widely varying rates. We noticed that *parE* reads were significantly more abundant than *gyrB* reads among families *Leuconostocaceae* (60 to 90%) and *Carnobacteriaceae* (70 to 95%) and in the genus *Brochothrix* (80%). However, within these taxa, the exact ratio between reads of *gyrB* and *parE* was species-dependent. For example, within the *Lactobacillaceae*, approximately 80% of reads assigned to *Lactobacillus algidus* represented *parE* sequences, while the same was true of only ~30% of the reads assigned to *Lactobacillus sakei*. Interestingly, with the exception of *Clostridium algidicarnis* for which the total number of reads remained below 50 in the whole dataset, the relative abundance of *gyrB* and *parE* sequences was relatively stable within each of the twenty *Firmicutes* species from one sample to another. Indeed, the standard deviation for these values from all *Firmicutes* species varied from 0 to ±13%. It can thus be hypothesized that, for a given species, the ratio between reads of *gyrB* and *parE* across samples is relatively constant. We also noticed that after chimera filtering none of the filtered OTUs were chimeras between *gyrB* and *parE*. Furthermore, the use of *gyrB* only induces the formation of 1.2% of chimera (corresponding to 10.6% of the clusters) while 16S rDNA generates 9.5% of chimera (corresponding to 17.9% of the clusters). Therefore, chimera formation was not a valid explanation for this phenomenon.

**Fig 3:**
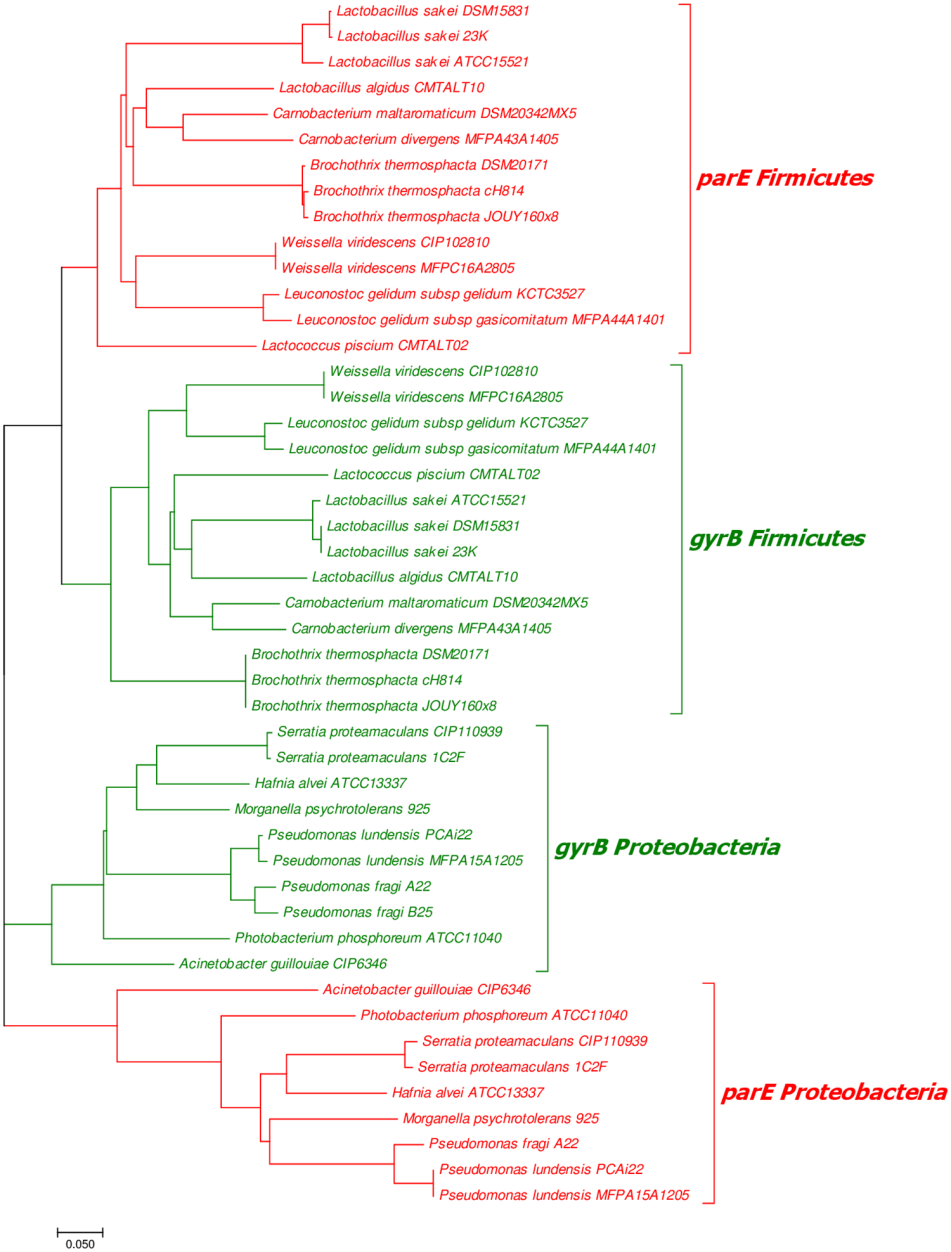
Unrooted phylogenetic tree constructed with the partial *gyrB* and *parE* sequences extracted from the genomes of bacterial species present in the mock communities. Branch length scale indicates the number of nucleotide substitutions per site. The tree is divided into three main branches: *parE* genes from *Proteobacteria*, *gyrB* genes from *Proteobacteria*, and *parE/gyrB* genes from *Firmicutes*. The two sub-branches discriminating between *parE* and *gyrB* genes from *Firmicutes* are equally distant from the *gyrB* branch of *Proteobacteria*.

We decided to further investigate the reason why amplification of *gyrB* also recovered *parE* genes only from *Firmicutes* and not from *Proteobacteria*. To do this, we performed a phylogenetic analysis that estimated the distances between *gyrB* and *parE* sequences of the species that were introduced in the mock communities (Fig 3). As we expected, *gyrB* sequences of different members of phylum *Firmicutes* appeared to be more similar to *parE* sequences from those same *Firmicutes* genomes than to *gyrB* sequences extracted from *Proteobacteria* genomes. This result confirms why the *parE* paralog gene was amplified only within strains belonging to phylum *Firmicutes* in our experiment.

These results highlight that, within ecosystems containing *Firmicutes* species, *gyrB* assignment databases must also take *parE* sequences into account. Here, both types of OTUs were included in all further analyses and are referred to collectively as *gyrB* sequences (in the context of comparison to 16S rDNA) unless a specific mention of *parE* sequences is necessary.

### Genus-level bacterial diversity in food is comparably described by 16S and *gyrB* amplicon analysis

**Fig 4:**
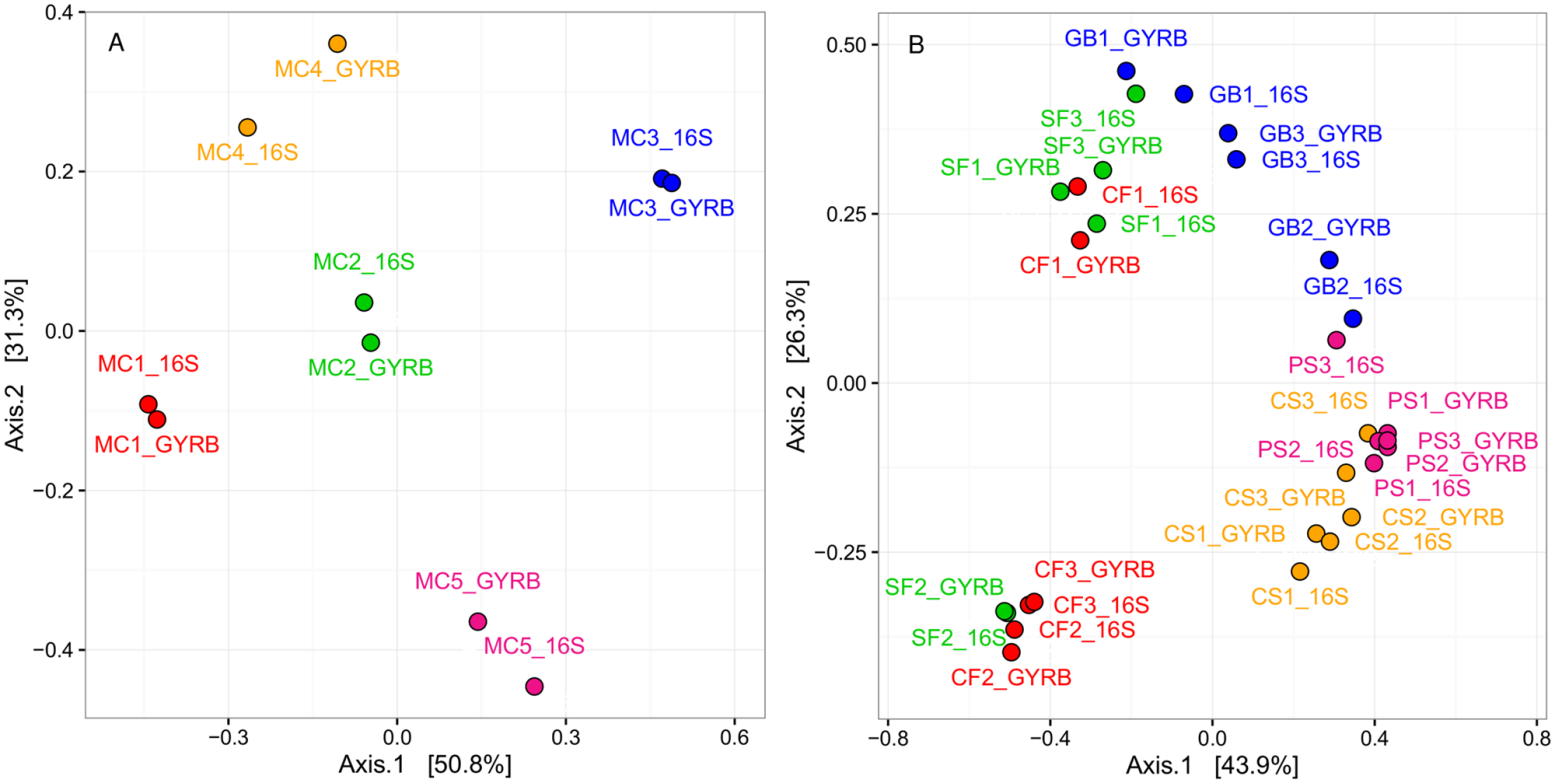
Principal coordinates analyses (PCoAs) based on Bray-Curtis distances among communities recovered by16S rRNA and *gyrB* gene sequencing of (A) mock communities and (B) food samples. All OTUs within the communities were identified to the level of genus. The first and second axes of the PCoA performed for MC samples explained a high degree of the influence of OTUs on communities, with respectively 50.8% and 31.3% of the total variance. Similarly, the first and second axes of the PCoA performed on food sample communities explained, respectively, 43.9% and 26.3% of the total variance.

Because the OTUs from both the 16S- and *gyrB*-based analysis could be accurately assigned to genus-level taxonomic identifications, we merged all OTU data to this level. We then performed principal coordinates analyses (PCoAs) based on Bray-Curtis distances to statistically compare the bacterial diversity detected using the two markers within the mock communities (Fig 4A) and food samples (Fig 4B). Both PCoAs revealed a clustering pattern that was strikingly similar between communities analyzed with *gyrB* and those analyzed with 16S rDNA. At the genus level, it thus appeared that *gyrB* and 16S had captured nearly identical images of the community composition for all samples.

In the PCoA of the mock communities, it is interesting to note that MC1, MC2, and MC3 are evenly distributed along an axis whose orientation is close to that of the first axis of the PCoA. Since these samples consisted of the same species (identical richness) mixed in different proportions, this axis could potentially represent the evenness between *Proteobacteria* species and *Firmicutes* species. Moreover, the second axis of the PCoA also appeared to correspond to the species richness found in our sample: while MC4—composed of only 5 genera—was located in the lower part of this axis, MC5—consisting of about 40genera—was found on the upper part of this axis. This trend was confirmed by the placement of MC2, which contained 15 distinct genera and which was located at an equal distance to both MC4 and MC5.

The PCoA of the mock communities provided a good view of the fidelity of *gyrB* with respect to 16S rDNA in standardized samples. The second PCoA likewise demonstrated that the two markers yielded similar results, but this time in the naturally occurring bacterial communities of food samples. Furthermore, this statistical analysis confirmed that any potential bias that may have been introduced due to variations in the prevalence of *parE* among the *gyrB* sequences was heavily outweighed by the structuring influence of food origin: in most cases, communities recovered from a given food product clustered together on the factorial plane regardless of the marker used for sequencing. There were, however, differences among samples of certain food types. For example, while the community composition of all samples of pork or poultry sausage was generally similar, samples of salmon and cod fillet yielded bacterial communities that were clearly more variable among themselves. Interestingly, the low Bray-Curtis distance between 16S- and *gyrB*-based pork sausage samples contradicted the results of the rarefaction analysis. As Bray-Curtis distance is sensitive to the abundance of shared OTUs, this difference might have stemmed from the prevalence of OTUs of very low abundance, which could have increased species richness in the 16S rDNA pork sausage samples compared to *gyrB* samples. A closer look at these OTUs revealed that 80% of the OTUs that comprised the increased richness in the 16S pork sausage samples were assigned to five genera (*Lactobacillus*, *Leuconostoc*, *Brochothrix*, *Pseudomonas*, and *Acinetobacter*) which are in fact the main constituents of the abundant microbiota in these samples. Therefore, we concluded that 16S amplification in the pork sausage samples had unexpectedly generated a high degree of artificial diversity which was not filtered out during our quality control to remove spurious OTUs.

**Fig 5:**
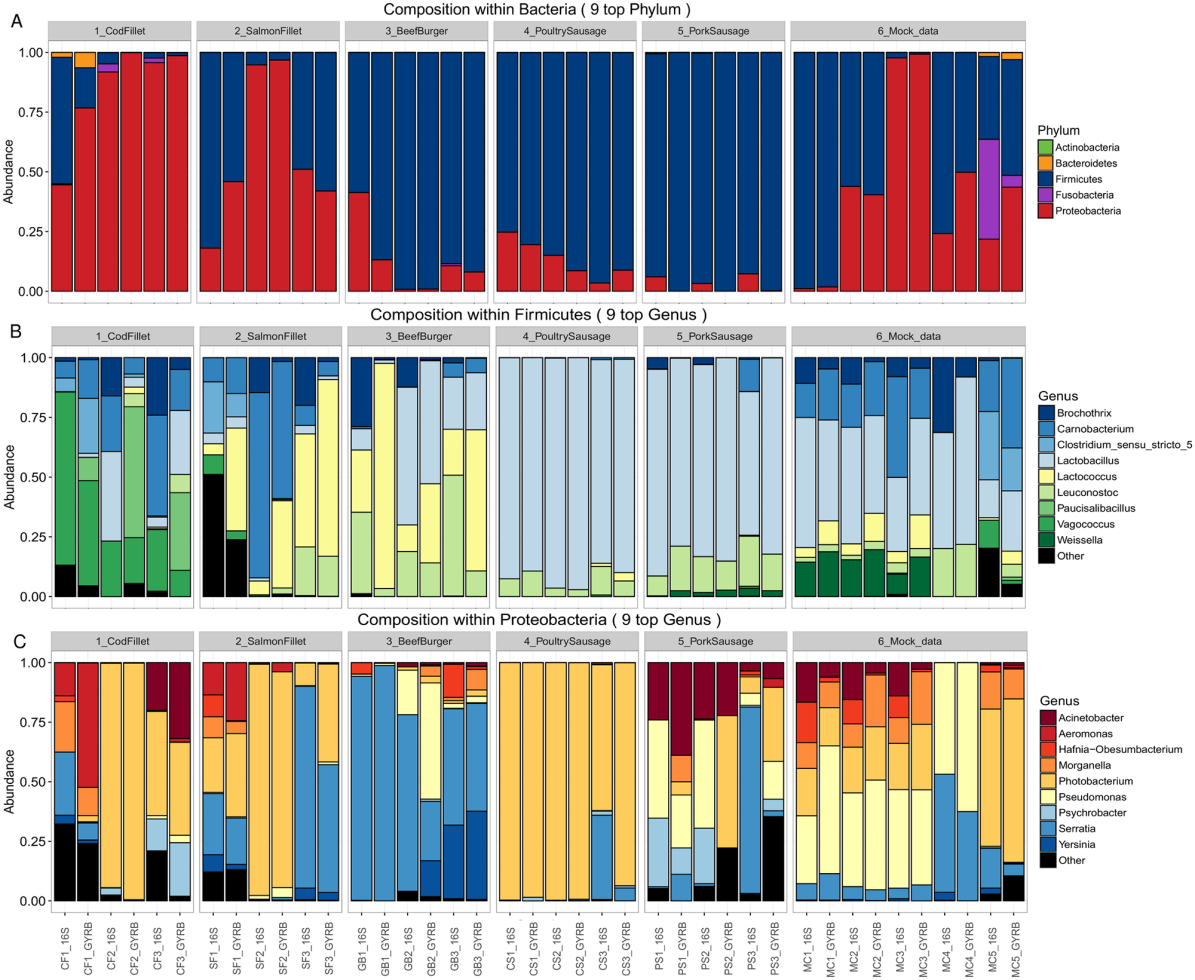
Composition plots of relative abundances of OTUs generated by 16S rRNA and *gyrB* sequencing (A) within *Bacteria* at the phylum level, (B) within *Firmicutes* at genus level, and (C) within *Proteobacteria* at genus level. Samples from a given food product are presented together (subpanels within A-C) and the two marker analyses for each sample are presented next to each other. Within each panel, the ratio of each taxon was estimated from the sum of all taxa (within all phyla, within *Firmicutes*, or within *Proteobacteria*, respectively).

The relative bacterial composition of each sample at both the phylum and genus levels is shown in Fig 5. As expected, all samples mainly consisted of members of two major phyla: *Firmicutes* and *Proteobacteria*. Interestingly, when the bacteria were viewed at the phylum level (Fig 5A), *gyrB* gene sequencing and 16S rRNA gene sequencing recovered highly comparable communities in 15 out of the 20 samples. Within these samples, the difference between 16S and *gyrB* gene sequencing in the proportion of sequences assigned to *Firmicutes* was 4.0±2.6%. A similar difference of 4.3±2.7% was calculated for the *Proteobacteria*. However, much larger differences between the two markers were noticed in five samples (CF1, SF1, GB1, MC4, and MC5). In MC5, this difference was due to the underestimation of sequences assigned to *Fusobacteria* by *gyrB* (5% versus 42% for 16S). It should be noted that 16S sequencing detected two different genera in this phylum: the first was the genus *Cetobacterium* (16S_cluster_60 and 16S_Cluster_377), which accounted for 2.5% of total bacterial abundance, while the second was an unknown genus (16S_cluster_41) affiliated with the putative *Hados*.*Sed*.*Eubac*.*3* family (SILVA nomenclature) that has been previously identified in cod fillet [1]. Here, this unknown genus accounted for 31.2% of the total bacterial abundance in MC5 and a minor abundance of about 1 to 2.7% in the cod fillet samples (CF1 to CF3). Although *Cetobacterium* was also detected with *gyrB* sequencing (*gyrB*_cluster_48 and *gyrB*_cluster_116) at a similar range of abundance (5.4%) in sample MC5, no OTU was identified for the second genus. Therefore, the bias was directly connected to the lack of amplification of this unknown genus. For the four other samples, no specific trend was evident: *gyrB* sequencing favored *Firmicutes* over *Proteobacteria* in CF1, SF1, and MC4, but favored *Proteobacteria* over *Firmicutes* in GB1. Furthermore, in MC4 the expected relative abundances of *Firmicutes* and *Proteobacteria* were 65% and 35%, respectively. However, we observed that the relative abundances obtained from the *gyrB*-based approach deviated from these expected values nearly as much as those from the 16S-based analysis did (*gyrB*: 50% *Firmicutes*, 50% *Proteobacteria*; 16S: 76% *Firmicutes*, 24% *Proteobacteria*). It was thus difficult to interpret biases in these data unless they were linked to the presence or absence of a specific genus in a given sample.

Within phylum *Firmicutes* (Fig 5B), the relative abundances of most genera were similar between the *gyrB*-and 16S-based approaches. For example, the differences between analyses were 1±1% for *Enterococcus*, 3±2% for *Weissella*, 5±5% for *Carnobacterium*, and no more than 7±7% for *Lactobacillus*. Nevertheless, we found that, using *gyrB*, sequences assigned to *Lactococcus* were recovered at higher abundances (e.g., +22% in GB2 up to +38% in SF1 and +68% in GB1) from food samples where this genus was abundant (GB and SF). Instead, this disparity was not confirmed in mock communities: although *Lactococcales gyrB* sequences were more abundant than the corresponding 16S rDNA sequences, the difference was much smaller, 6±1%. It is probable that the large differences in *Lactococcus* abundances within GB and SF samples are related to the fact that the *gyrB*-based analysis assigned a lower number of reads to *Brochothrix* and *Leuconostoc*. Indeed, in CS, PS, and MC samples the average difference between the two markers in terms of reads assigned to *Leuconostoc* was 4±3%; instead, in GB1 and GB3, 16S reads were 4.5–7.0 times more abundant. Similarly, within all samples, the 16S analysis assigned up to 85 times more reads to *Brochothrix* than the *gyrB* analysis did. It is mainly notable within CF (CF2 and CF3), SF (SF2 and SF3) and MC (MC4) samples.

Within the *Proteobacteria* (Fig 5C), the three PS samples could not be analyzed because too few sequences were assigned to this phylum by *gyrB* (18, 9, and 176 sequences for PS1, PS2, and PS3 respectively). As we found with the *Firmicutes*, *Proteobacteria* community composition was largely similar between both analyses. The average difference across all genera in relative abundance between 16S and *gyrB* sequences was 6±9%. However, some differences were noted in a few individual samples and for certain genera. For instance, in samples such as CF1, SF3, GB2, and CS3, the number of sequences assigned to *Serratia* was on average 2.4±0.4 times higher with 16S than with *gyrB*. In SF3 and CS3, the relative abundance among genera of *Proteobacteria* of *gyrB* sequences assigned to *Photobacterium* was 41% and 94%, respectively, while these values dropped to 9% and 61% for the same samples using 16S. In general, we observed that the discrepancies between the two markers increased for subdominant members of a population, who, due to their low abundances, may have suffered from a decline in sequencing quality. Furthermore, we found that some of the slight discrepancies between *gyrB* and 16S sequencing in terms of the abundance of genera from the *Enterobacterales* (such as were observed in the mock communities) were due to incorrect taxonomic assignment of the 16S OTUs by the pipeline. For instance, 3.4% of 16S reads from sample MC4_16S were assigned to genus *Yersinia* in the *Enterobacterales*; however, from this bacterial family, MC4 contained only *Serratia proteamaculans*.

### Quantification biases between *gyrB* and 16S rRNA genes are not significantly different

**Fig 6:**
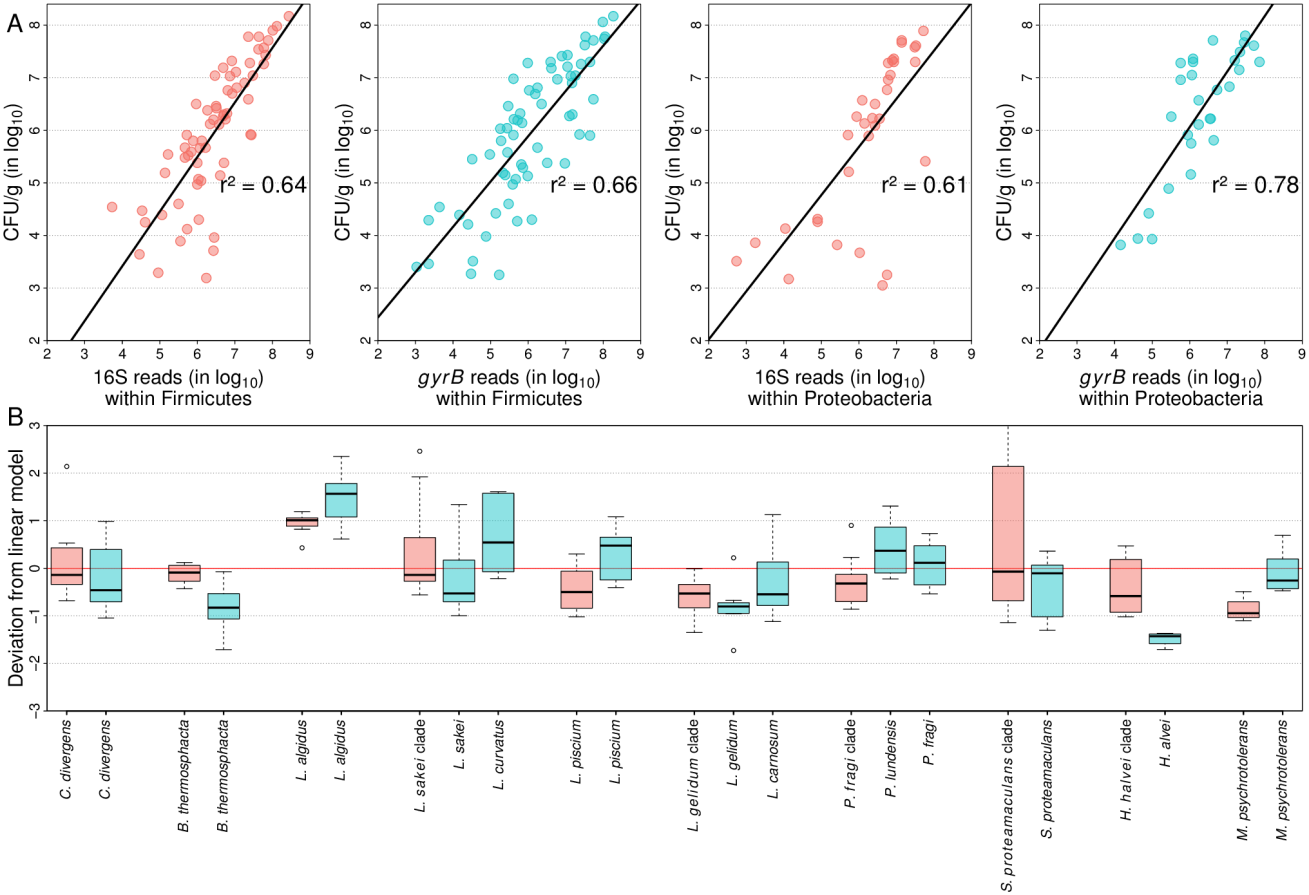
Comparative analysis of quantification of bacterial species by qPCR versus the estimated absolute number of reads obtained by amplicon sequencing. **(A)** Results obtained with 16S rDNA and *gyrB* amplicon sequencing are shown in the upper plots (in pink and cyan, respectively) with data separated by phylum: *Firmicutes* (left) and *Proteobacteria* (right). **(B)** Boxplot showing the deviation from the linear regression model of the quantification obtained with 16S rDNA (pink) or *gyrB* (dark cyan) for several bacterial species in the food samples. For the purpose of quantification of the 16S rDNA data, species were merged to genus or broad infra-genus phylogenetic clades because the obtained OTUs could only be assigned to these levels (see main text on subspecies-level bacterial richness).

We next quantified all 13 out of the 16 species that constituted the mock communities in order to investigate two issues: first, the existence of possible biases in the quantification of some taxa, and second, the ability of *gyrB* to yield quantitative estimates of the main bacterial species in food products that were at least as reliable as those obtained using 16S rDNA. To do this, we used quantitative PCR to estimate the total bacterial population in CFU.g^-1^ using a universal primer set (S1 Table). The total concentration of bacterial cells was then used to convert the relative read counts of each taxon into extrapolated estimations of the species concentrations in absolute read counts (see Materials& methods section). The estimated absolute number of16S reads and *gyrB* reads were thus compared separately to the qPCR results, which were expressed in CFU.g^-1^ at the taxonomic level of phylum as described in Fig 6.

A linear regression model was constructed for each of the four datasets obtained from food samples and mock communities (*Firmicutes* 16S, *Firmicutes gyrB*, *Proteobacteria* 16S, and *Proteobacteria gyrB*). For all tested species, there was no evidence of additional bias in the comparison between qPCR results and *gyrB* reads with respect to the comparison between qPCR and 16S rDNA. However, some discrepancies in quantification between qPCR and amplicon sequencing were found for both markers; these inconsistencies appeared to be linked with the presence of rare species in certain samples, indicating that either qPCR quantification or amplicon sequencing was more difficult or was subject to inhibitory effects for less-abundant species. Furthermore, the r^2^ values of the linear regression models that correlated qPCR results with either 16S rDNA or *gyrB* read counts were similar for species in phylum *Firmicutes* but significantly higher for *gyrB* reads for *Proteobacteria* species. These results thus confirm that analyses based on this gene are better able to faithfully represent the composition of complex bacterial communities in food products.

For each species, we estimated the deviation between the number of experimental reads and the number of theoretical reads calculated by the linear regression model. For 11 out of the 13 species, the median deviation was inferior to 1.0 log_10_(Fig 6B), thus confirming that *gyrB* can be used alongside 16S to successfully determine the composition (richness and evenness) of food microbiota.

For *Lactobacillus algidus*, amplicons of both genes appeared to overestimate its proportion within the ecosystem. However, the strong correlation between the performances of both genes could indicate the possible presence of a quantification bias in the qPCR method(perhaps a specific inhibition of *L*. *algidus* primers), or a possible specificity problem of these primers(originally designed of strain CMTALT10 GA [33], which might be unrepresentative of the strains present in the food samples). Instead, *gyrB* appeared to slightly but systematically underestimate the abundance of *Hafnia alvei* and *Brochothrix thermosphacta* compared to 16S rDNA.

### Accuracy of *gyrB*/*parE*-based OTUs in determining subspecies-level bacterial richness

**Fig 7:**
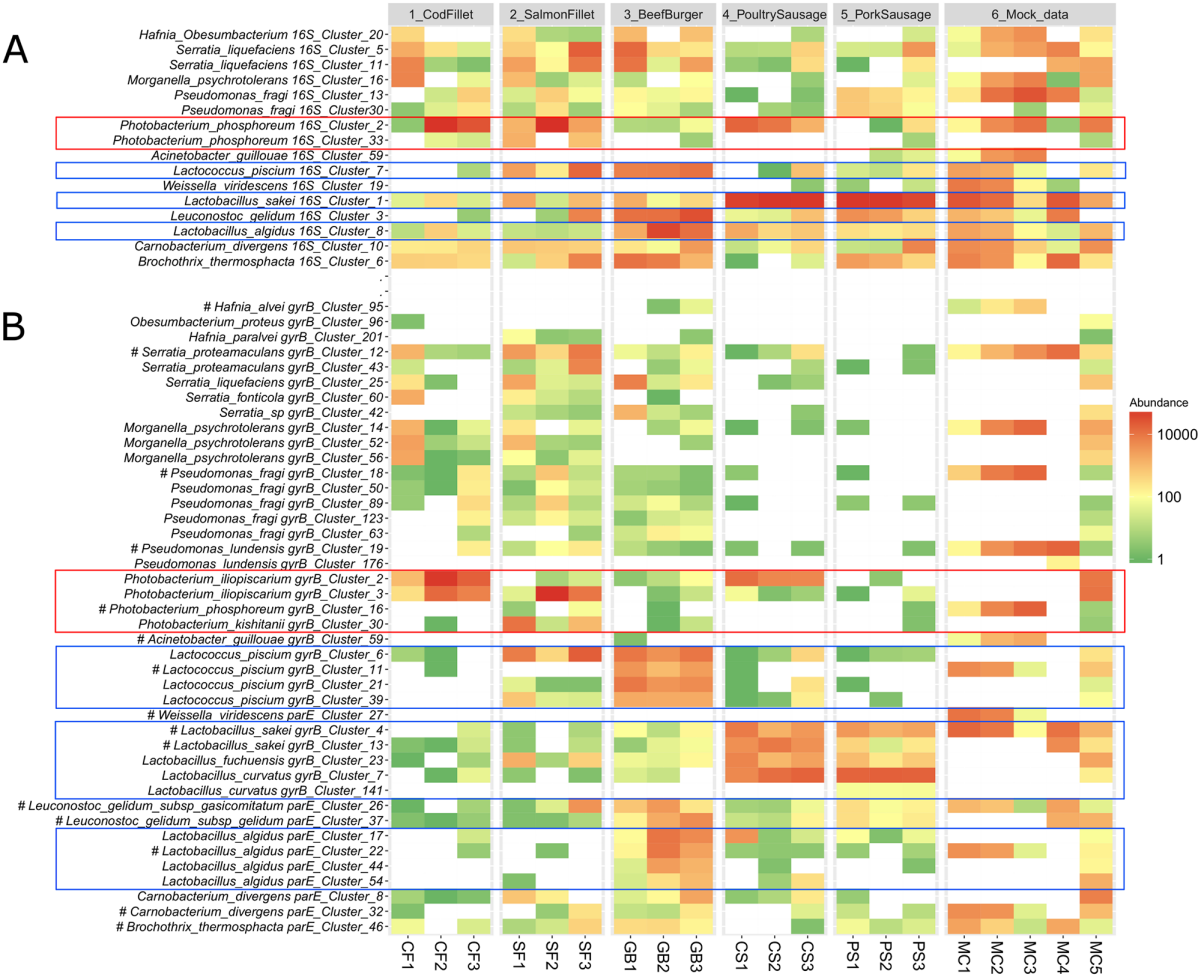
Heatmap showing the ability of (A) 16S rDNA and (B) *gyrB* amplicon analysis to estimate intraspecies population levels. Samples are ordered from left to right according to the sample type. The scale on the right of the heatmap depicts the color palette associated with the relative numbers of reads of the various OTUs. OTUs are labeled with their cluster number and the taxonomic assignment at the species level. The *gyrB/parE* OTUs associated with the strains used in the mock communities are labeled with (#). Boxes are drawn around the main phylogenetic clades that are detailed in the text.

**Fig 8:**
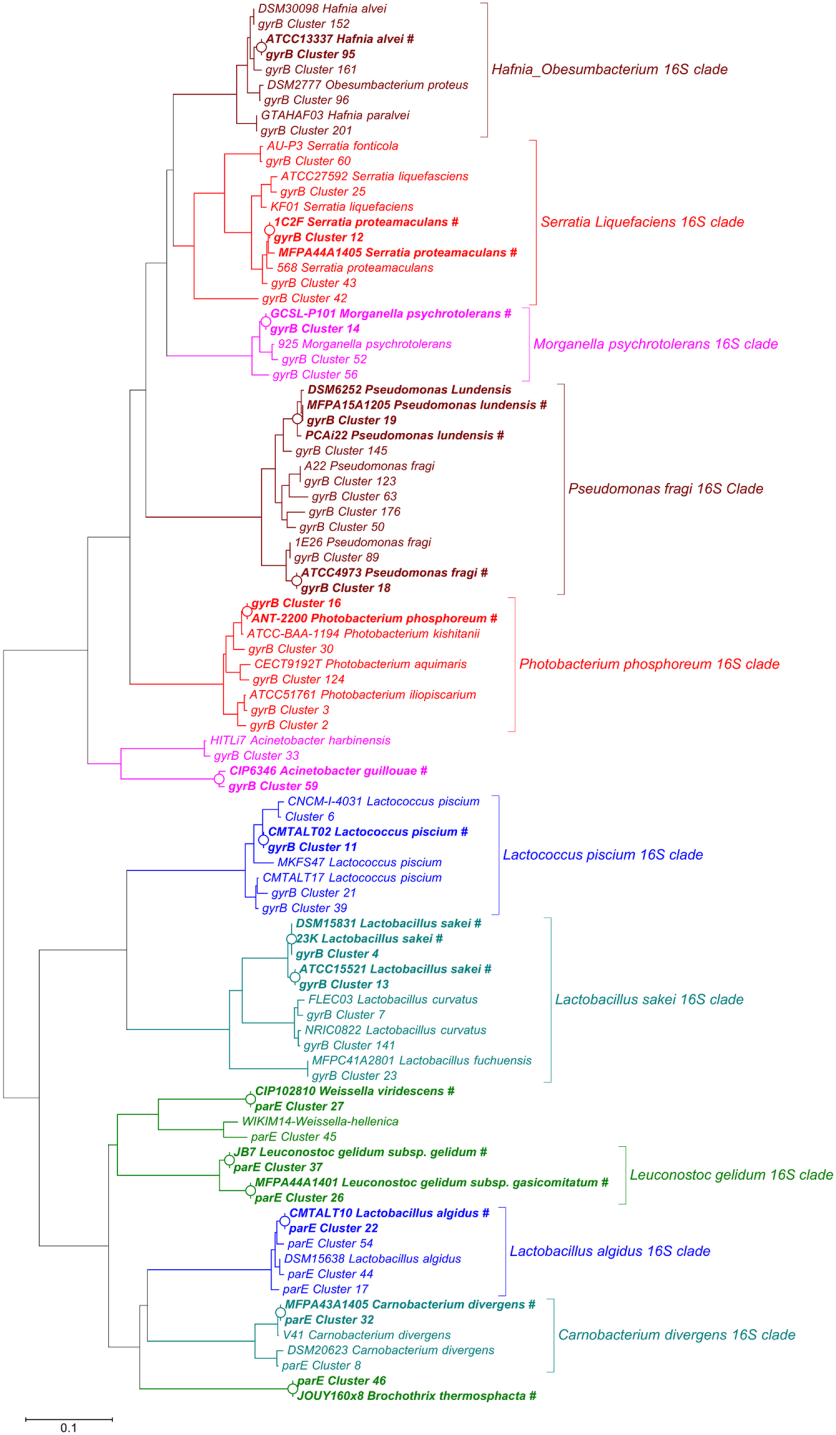
Unrooted neighbor-joining phylogenetic tree showing the evolutionary relationship between *gyrB* or *parE* sequences from OTUs in the current study and those from the published genomes of sequenced strains. OTUs are labeled with their cluster numbers; sequences extracted from published genomes are labeled with the strain name followed by the species name. Strains used in the mock communities are indicated in bold type and tagged with (#). The tree nodes where OTUs and strain sequences cluster are indicated by an open circle (O). Brackets drawn on the right of major *gyrB*/*parE* clades indicate the identities of the corresponding 16S phylogenetic clades.

To investigate the discriminatory power of using *gyrB* at the subspecies level, we further focused on the 10 most-abundant phylogenetic clades recovered by 16S rDNA amplicon sequencing, which comprised~80% of the reads in the 16S dataset. As mentioned in Fig 6, we refer here to phylogenetic clades because most of the thirteen 16S-based OTUs (named 16S_Cluster_XX, for example) representing these 10 clades could not be assigned a species-level taxonomic identification. Instead, we defined our OTUs for this analysis as groups of phylogenetically related species that cluster together at a threshold of 97% identity; these clades thus represent an intermediate situation between genus and species. To these ten clades, we added three additional genera (*Brochothrix*, *Weissella*, and *Acinetobacter*) because they had been included as controls in the mock communities (MC1 to MC4). We then constructed a heatmap that showed the abundance of these sixteen OTUs assigned through 16S rDNA amplicon sequencing of the food and mock communities samples (Fig 7A). These phylogenetic clades corresponded to 44 *gyrB*-based OTUs (named, e.g., *gyrB*_Cluster_XX), for which a heatmap was also constructed (Fig 7B). To facilitate comparison between these two analyses, a phylogenetic tree was constructed that included all *gyrB*-based OTUs along with the *gyrB* or *parE* sequences (extracted from public databases) of the different species and strains identified in the various samples or used in the mock communities (Fig 8). In general, it appeared that the *gyrB*-based approach was able to resolve broad phylogenetic clades to the species-or subspecies-level with a high degree of accuracy. There were two notable examples of this. First, using *gyrB*, the 16S-based clade of *Photobacterium phosphoreum* was resolved into four species (*P*. *phosphoreum*, *P*. *iliopiscarium*, *P*. *kishitanii*, and *P*. *aquimaris*), which gave us the ability to detect each of these species in the various samples. For example, *P*. *iliopiscarium* was revealed to be the most prevalent species in the food samples. Interestingly, poultry sausages were contaminated with a strain (*gyrB*_cluster_02) that was phylogenetically distinct than the one found in salmon fillets (*gyrB*_cluster_03). Instead, *P*. *phosphoreum* (the species itself, *gyrB*_cluster_16) was only found in the mock communities (MC1 to MC3) in which it had been introduced, as a strain isolated from spoiled fish, on purpose. The second example of the discriminatory power of *gyrB* was provided with the broad *Lactobacillus sakei* clade, which in the *gyrB*-based analysis was separated into three species (*L*. *sakei*, *L*. *curvatus*, and *L*. *fuchuensis*). Through this improved resolution, we were able to see that both poultry and pork sausages were rather contaminated by *L*. *curvatus* and not by *L*. *sakei*, on the contrary to the results of16S-based analysis. Likewise, *gyrB* added additional details to the analysis of *L*. *sakei* strains in mock community MC4 (one-third of abundance for *gyrB*_cluster_13 corresponding to strain DSM20017 and two-thirds of abundance for *gyrB*_cluster_04 corresponding to strains 23K and DSM15831grouped together). Two final examples which strengthen this point come from *Lactococcus piscium* and *Lactobacillus algidus*. Both of these species was identified at the species level with 16S-based OTUs, but the analysis of *gyrB* detected diversity at the subspecies level, with up to four different *gyrB*-based OTUs in each species. Of these, only one OTU per species (*gyrB*_cluster_11 for *L*. *piscium* and *gyrB*_cluster_22 for *L*. *algidus*) specifically matched the strain that had been added in the mock communities, with the other strains coming from the various food samples. Instead, the opposite case was found for *Brochothrix thermosphacta*: this species had only one corresponding *gyrB*-based OTU, corroborating earlier reports that this species has a very limited, almost clonal population structure [63].

## Discussion

The aim of the present work was to assess the utility of *gyrB* amplicon sequencing for analysis of the bacterial diversity of food microbiota at the subspecies level; specifically, we wanted to investigate the performance of this marker relative to that of the 16S rDNA V3-V4 region, which is commonly used for this purpose. The impetus for this study arose from the need to decipher this type of microbiota at a subspecies level.

Several previous studies performed with the *gyrB* gene showed promising results regarding the use of this marker for analysis of microbiota diversity. However, these studies had focused on the microbiota of plant seeds (which is mainly composed of *Proteobacteria*) or on the *Bacteroidetes* phylum of the human gut microbiota. The potential of *gyrB* amplicon sequencing for analyzing the intraspecies diversity of food microbiota thus remained to be assessed.

Our results demonstrate that *gyrB* sequencing can fulfill this goal. This housekeeping gene shows around 94 to 95% sequence identity among strains of the same species, a level of variation that matches the ANI (Average Nucleotide Index) value now commonly used for species-level estimation [34]. Therefore, binning reads at 97% or 98% identity, as typically occurs in OTU clustering, offers the possibility to capture intraspecies diversity within the main lineages. This ability to distinguish among groups of phylogenetically distinct strains (population lineages, main clonal complexes, and so forth) has enormous implications for our knowledge of the bacterial strains and population fluctuations involved in food processes. Furthermore, with an increase in the number of sequenced strains from food-borne bacteria, *gyrB* amplicon sequencing will help to better infer functional profiles of diverse microbiota using predictive tools such as PICRUST or Tax4Fun.

The relative quantification of species using *gyrB* did not yield different or even better results than that obtained with 16S amplicon sequencing, despite the unexpected amplification of the *parE* gene from *Firmicutes* species. Within this phylum, these genes, which encode topoisomerases II and IV, are very closely related and are co-amplified in a manner that is strongly species-dependent. This phenomenon had not been previously observed because the degenerate primers from the study of Barret et al. 2012 [21]had only been previously tested in microbiota exclusively composed of *Proteobacteria* (in plant seeds). The similarity of *gyrB* and *parE* nucleotide sequences in *Firmicutes*, and in particular in the order *Lactobacillales*, significantly complicates the task of designing better universal primers. One solution could be to design several sets of primers for each major taxonomic level (phyla or orders), as was performed in the human microbiome study [24]. Even this approach, though, cannot be guaranteed to prevent the unwanted amplification of *parE* from some species (notably, within families *Leuconostocaceae* and *Carnobacteriaceae*). We noticed that for the species in which *parE* reads represented a significant proportion of recovered amplicons (>50%), relative quantification was slightly underestimated (around 10%) compared to the absolute quantification measured in CFU.g^-1^. Therefore, the quantitative estimates based on *gyrB* amplicon sequencing were less reliable for *Firmicutes* species compared to estimates based on 16S rDNA amplicon sequencing. A further challenge will be the need to include in a computing pipeline two different databases (*gyrB* and *parE*) for taxonomic assignment.

From our point of view, however, these two caveats are minor problems which are far outweighed by the major advantages of *gyrB* sequencing in improving species-level taxonomic assignment and in investigating OTU richness at the subspecies level. Here, this improvement was clearly shown in the analysis of phylogenetic clades that are known to contain several closely related species and lineages, such as *Photobacterium phosphoreum*, *Serratia proteamaculans*, *Pseudomonas fragi*, and *Lactobacillus sakei*. Our comparative analysis also highlighted how taxonomic assignment based on 16S sequencing yielded erroneous results within some bacterial orders such as the *Enterobacterales*. Food microbiota often contain groups of genera that are closely phylogenetically related, such as *Serratia*, *Hafnia*, *Yersinia*, and *Morganella*. Artificial diversity, which is created inherently by the 16S amplification process (PCR, sequencing, etc.), may generate drift up to 3% of sequence identity. In this particular clade of *Enterobacterales*, this degree of variation was sufficient to switch a putative identification from one genus to another. Thus, the quantification of *Enterobacterales* and of *Proteobacteria* in general, was greatly improved with *gyrB* sequencing.

Another major benefit of *gyrB* sequencing is the ability to capture OTU richness at the subspecies level. Our data, in particular those shown in Fig 1, clearly demonstrate how subspecies diversity can influence the image of richness within a sample in comparison to an analysis that misses this diversity (i.e. the 16S strategy). In brief, when diversity at the subspecies level is low, *gyrB* and16S rDNA sequencing strategies will recover similar OTU richness, but the gap between these approaches will broaden as the subspecies diversity increases (see, for instance, mock community MC5). Thus, one clear benefit of *gyrB* is to improve analyses of beta diversity by enabling more-accurate discrimination of samples. In particular, analyses based on this marker would also facilitate comparative studies of the beta diversity of microbiota that contain a limited number of different species but in widely varying abundances. In this point of view, the sequencing of many strains among spoilage species will facilitate the estimation on how *gyrB*-based intra-species diversity could be assessed. In addition, the use of *gyrB* allows to get rid of unspecific amplification of mitochondrial or chloroplastic 16S rDNA often detected at high percentage (up to 80%) in food products of animal origin supplied with spices when 16S V1-V3 region is used [1].

The final point we would like to highlight is the fact that *gyrB* sequence diversity is most likely species dependent. Therefore, the ability of *gyrB* amplicon sequencing to reveal accurately subspecies-level diversity may vary significantly among species. Our work revealed a clear example of this particular problem: only one *gyrB* cluster was identified for *B*. *thermosphacta*, a species that has been recognized as lacking strong intraspecies diversity [63].Instead, up to four clusters were identified in *L*. *piscium* or *P*. *fragi*. An additional complication is that the bacterial DNA gyrase is the target of some antibiotics [64], and the selective pressure created by the long exposure of some pathogenic bacteria to antibiotic treatment has been shown to induce mutant variants of the *gyrB* gene. This factor must be taken under consideration if *gyrB* amplicon sequencing is to be used, for example, for pathobiome analysis.

## Conclusions

In sum, our opinion is that *gyrB* sequencing would be very valuable in analyses of bacterial diversity that are specifically directed at deciphering details of population structure at the subspecies level. This approach would carry notable benefits for the temporally and/or spatially extensive campaigns that are often carried out on food microbiota, e.g., studies that track and trace whether particular subspecies lineages are specifically selected or subjected to seasonal changes within a food production chain or during the shelf life.

However, we believe that 16S rDNA amplicon sequencing should still be incorporated in these metagenetic analyses as a control (by selecting a subset of samples for instance) in order to ensure that the *gyrB* data remain consistent with those of the universally used 16S rDNA. Therefore, we would not recommend the use of *gyrB*-based methods to *de novo* analyze microbiota that are completely unknown; indeed, our data showed that some species that are not yet well characterized (e.g., the unknown genus found in cod fillet) might be missed. Generally speaking, *gyrB* sequencing still needs to be tested in many different types of complex microbiota and especially in those that contain phyla other than *Firmicutes* and *Proteobacteria*.

## Supporting information

S1 Table Specificity and nucleotidic sequence of the primers used in quantitative PCR. (DOCX)

S1 Fig Comparative plot showing the relationship between bacterial OTUs and genera for both 16S rDNA and *gyrB* sequencing. (PDF)
