# Supplementary material for: Deciphering intra-species bacterial diversity of meat and seafood spoilage microbiota using gyrB amplicon sequencing: A comparative analysis with 16S rDNA V3-V4 amplicon sequencing
Source: PLoS One. 2018 Sep 25;13(9):e0204629. doi: 10.1371/journal.pone.0204629 (PMC6155546; doi:10.1371/journal.pone.0204629)
Supplement: S1 Table — (DOCX) [file pone.0204629.s001.docx]

| Primer pair | Target species  (target gene) | Sequence (5'-3') | Amplicon size (bp) | Annealing temp (°C) | Reference |
| --- | --- | --- | --- | --- | --- |
| QSF03-BTH-F | *Brochothrix thermosphacta* (*rpoC*) | GGACCAGAGGTTATCGAAACATTAACTG | 148 | 58 | [1] |
| QSF03-BTH -R |  | TAATACCAGCAGCAGGAATTGCTT |  | 54 |  |
|  |  |  |  |  |  |
| QPVS05-CDI-F2 | *Carnobacterium divergens* (*rpoA*) | CTTGCATAACCTCTTCCTGATTTT | 244 | 52 | [1] |
| QPVS05-CDI-R2 |  | TGATGGCGTTGTTGAGGATGTAACT |  | 56 |  |
|  |  |  |  |  |  |
| QCMT06-LAL-F | *Lactobacillus algidus* (*recA*) | GGGTATTTTATCGTTGGATTACGCATTA | 120 | 56 | This study |
| QCMT06-LAL-R |  | ACCACCATTTTTTTGCACTTCTGCAGTC |  |  |  |
|  |  |  |  |  |  |
| QPVS06-LCU-F | *Lactobacillus curvatus* (*katA*) | CTGGTAAGAAAACACCAATGATTGCG | 123 | 56 | [1] |
| QPVS06-LCU-R |  | TCGTAGTTGCCTTCTTCGGTATAGAAC |  | 58 |  |
|  |  |  |  |  |  |
| QMF01-F | *Lactobacillus sakei* (*katA*) | CTGGCTATCCCGATACATACC | 186 | 54 | [2] |
| QMF01-R |  | GCATATCTTGGCTACGGGCA |  | 54 |  |
|  |  |  |  |  |  |
| QCMT05-LPI-F | *Lactococcus piscium* (rpoA) | TGAAGAAAATGTCTATGGTAAATTTGTCA | 140 | 53 | [1] |
| QCMT05-LPI -R |  | GAGTACACCGTCAATTTGGATGCTT |  | 56 |  |
|  |  |  |  |  |  |
| QPVS-05LGE-F | *Leuconostoc gelidum* (*rpoA*) | AGAACGAAGCCTTGAAGTTGATA | 115 | 52 | [1] |
| QPVS-05LGE-R |  | CTGCTACGGTTGCAATATGTAG |  | 53 |  |
|  |  |  |  |  |  |
| QPVS05-LCA-F | *Leuconostoc carnosum* (*rpoA*) | GATTGATTCAGATGAGGAACGTGTT | 84 | 54 | [1] |
| QPVS05-LCA-R |  | CGCACTGCTTGTAAATCAGCT |  | 55 |  |
|  |  |  |  |  |  |
| QCMT04-PLU-F | *Pseudomonas lundensis* (*rpoB*) | ATTGCCTGCGTCCGTGCTT | 180 | 53 | This study |
| QCMT04-PLU-R |  | ATCACTTTGCCCTTGTCATCCA |  | 53 |  |
|  |  |  |  |  |  |
| QCMT04-PFR-F | *Pseudomonas fragi* (*rpoB*) | GCTGCCTGCATCGGTATTG | 180 | 53 | This study |
| QCMT04-PFR-R |  | ATCACCTTGCCTTTGTCATCGG |  |  |  |
|  |  |  |  |  |  |
| QCMT04MPS-F | *Morganella psychrotolerans*(*rpoB*) | CCGGGAAAGTGGTTGCGCGT | 131 | 58 | This study |
| QCMT04MPS-R |  | CAGGGTTTCAATGGTTTTGTGACCG |  |  |  |
|  |  |  |  |  |  |
| QEBPSPR01-F | *Serratia proteamaculans*(16S) | CAGAATTCGGCAGAGATGCCTTA | 150 | 55 | This study |
| QEBPSPR01-R |  | TCCTTTGAGTTCCCACCATTACGT |  | 56 |  |
|  |  |  |  |  |  |
| QSF01HAL-F | *Hafnia alvei* (16S) | TAACTTGGGAACTGCATTTGAAACTGGTC | 110 | 59 | This study |
| QSF01HAL-R |  | CGCCACTGGTGTTCCTCCAGATC |  | 61 |  |
| F1369 | All Bacteria (16S) | CGGTGAATACGTTCYCGG | 220 | 53 | [3] |
| R1492 |  | GGWTACCTTGTTACGACTT |  | 53 |  |

S1 Table

REFERENCES

1. Fougy L, Desmonts MH, Coeuret G, Fassel C, Hamon E, Hezard B, et al. Reducing Salt in Raw Pork Sausages Increases Spoilage and Correlates with Reduced Bacterial Diversity. Applied and environmental microbiology. 2016;82(13):3928-39.

2. Chaillou S, Christieans S, Rivollier M, Lucquin I, Champomier-Verges MC, Zagorec M. Quantification and efficiency of Lactobacillus sakei strain mixtures used as protective cultures in ground beef. Meat science. 2014;97(3):332-8.

3. Sen B,Hamelin J, Bru‐Adan B, Godon JJ, Chandra TS. Structural divergence of bacterial communities from functionally similar laboratory‐scale vermicomposts assessed by PCR‐CE‐SSCP. J. Appl. Microbiol. 2008;105: 2123-2132
