# Supplementary figures and images for: Deciphering intra-species bacterial diversity of meat and seafood spoilage microbiota using gyrB amplicon sequencing: A comparative analysis with 16S rDNA V3-V4 amplicon sequencing
Source: PLoS One. 2018 Sep 25;13(9):e0204629. doi: 10.1371/journal.pone.0204629 (PMC6155546; doi:10.1371/journal.pone.0204629)

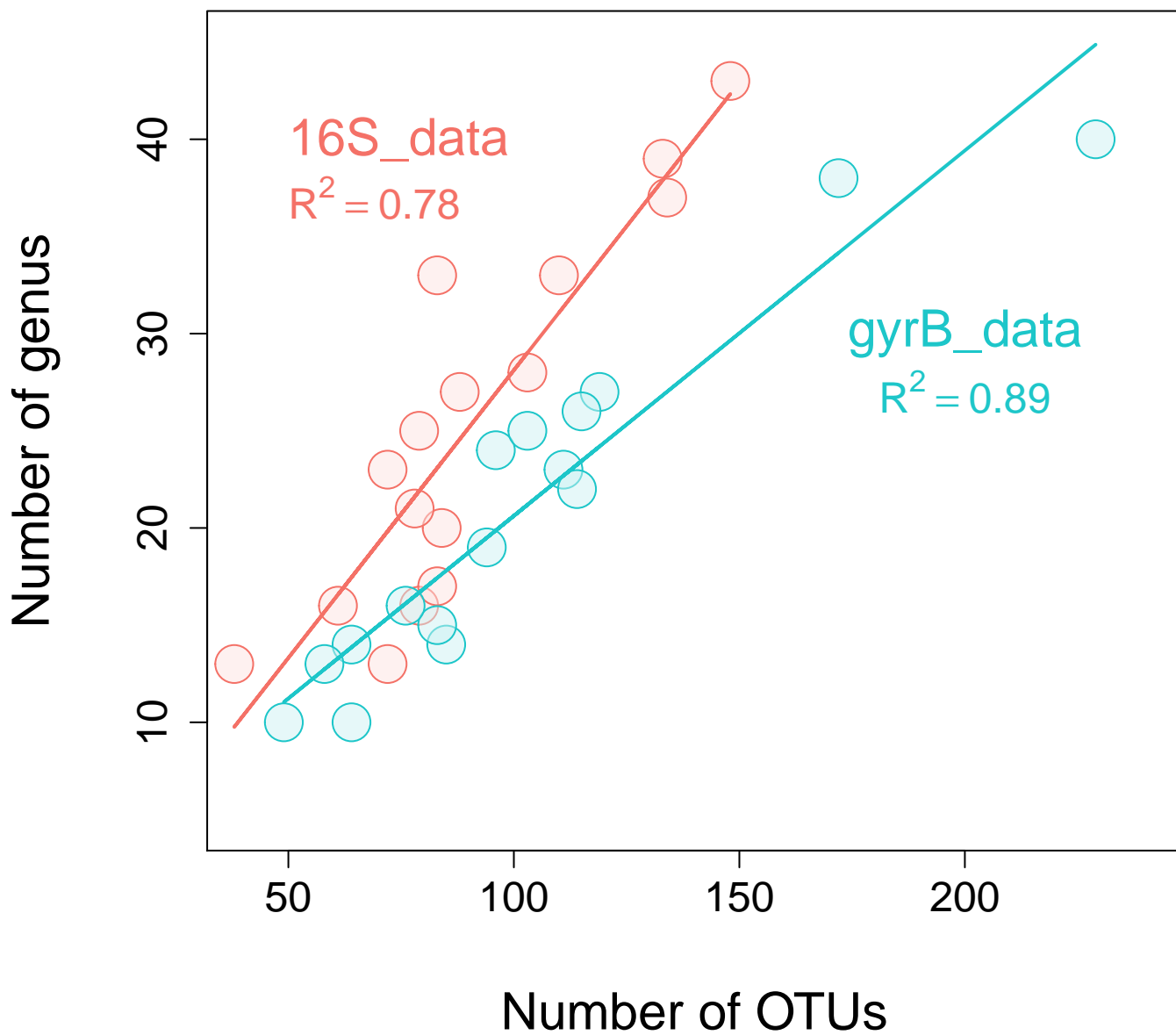

S1 Fig

Supplement: S1 Fig — (PDF) [file pone.0204629.s002.pdf]
